# GUT MICROBIOTA IN INFANTS WITH COW MILK ALLERGY: A SYSTEMATIC REVIEW OF CONTROLLED STUDIES

**DOI:** 10.1590/S0004-2803.24612025-133

**Published:** 2026-08-14

**Authors:** Marcela Duarte de Sillos, Juliana Satomi de Souza Matsuo, Mauro Batista de Morais

**Affiliations:** 1 Universidade Federal de São Paulo (UNIFESP), Departamento de Pediatria da Escola Paulista de Medicina (EPM), Médica Assistente da Disciplina de Gastroenterologia Pediátrica, São Paulo, SP, Brasil.; 2 Universidade Federal de São Paulo (UNIFESP), Departamento de Pediatria da Escola Paulista de Medicina (EPM), Médica Residente da Disciplina de Gastroenterologia Pediátrica, São Paulo, SP, Brasil.; 3 Universidade Federal de São Paulo (UNIFESP), Departamento de Pediatria da Escola Paulista de Medicina (EPM), Professor Sênior, Titular e Livre-docente pela Disciplina de Gastroenterologia Pediátrica, São Paulo, SP, Brasil.

**Keywords:** Microbiota, microbiome, food hypersensitivity, cow milk allergy, cow milk hypersensitivity, food protein induced, proctocolitis, hematochezia, Microbiota, microbioma, hipersensibilidade alimentar, alergia ao leite de vaca, hipersensibilidade ao leite de vaca, induzida por proteína alimentar, proctocolite, hematoquezia

## Abstract

**Background:**

Alterations in the gut microbiota may be involved in the pathophysiology of cow milk allergy (CMA). However, whether gut microbiota abnormalities contribute to the diagnostic confirmation of CMA through specific microbiome signatures is still unknown.

**Objective:**

To conduct a systematic review of the literature on the gut microbiota of infants with CMA.

**Methods:**

This systematic review included studies on the gut microbiota of infants aged <2 years with CMA at diagnosis and at follow-up after different interventions to control clinical manifestations and compared them with that of healthy controls. The PubMed database was used for literature search. The Preferred Reporting Items for Systematic Reviews and Meta-Analyses protocol was applied. This review was registered on the PROSPERO platform (CRD42024574354).

**Results:**

A total of 1,096 articles were identified. After applying inclusion and exclusion criteria, 18 studies were selected for the systematic review. Clinical manifestations included infants with immunoglobulin E (IgE)-mediated CMA (n=7), those with non-IgE-mediated CMA (n=10), or both (n=1). An oral challenge test for CMA diagnosis was mentioned in 11 studies, and in seven of them, a double-blind placebo-controlled challenge test was used. Most studies (n=13) used 16S rRNA gene sequencing to investigate the intestinal microbiota, and only three studies used shotgun metagenomic analysis. There was significant heterogeneity in the expression of results on microbiota characteristics. Alpha diversity was similar in the control group in most studies. A low abundance of *Bifidobacteria* was observed in some studies (n=5).

**Conclusion:**

The results of this systematic review did not identify a typical microbiota pattern in infants with CMA. Studies including infants before elimination diet and with a diagnosis confirmed by an oral challenge test, and studies including one group of infants of the same age on exclusive breastfeeding and another group of infants of the same age on formula feeding as a control group are needed. Therefore, currently available data do not allow CMA diagnosis through a microbiota signature.

## HIGHLIGHTS

• This study aimed to conduct a systematic review of the literature on the gut microbiota of infants with cow milk allergy.

• We included studies on the gut microbiota of infants aged <2 years with cow milk allergy at diagnosis and at follow-up after different interventions in controlling clinical manifestations and compared them with that of healthy controls.

• Eighteen studies were selected for systematic review after applying inclusion and exclusion criteria. There was significant heterogeneity in age of inclusion in the studies, diet consumed, diagnostic criteria for cow milk allergy, methods for analyzing the gut microbiota, timing of stool analysis, different hypoallergenic formulas during elimination diet, and heterogeneity in the expression of microbiota characteristics, thereby hindering interpretation of results. Significant heterogeneity was observed in the expression of results on microbiota characteristics. Alpha diversity was similar in study group and in the control group of most studies. Allergy to cow milk was associated with low involvement of *Bifidobacteria* in some studies.

• Therefore, currently available data do not allow the diagnosis of cow milk allergy to be established through a microbiota signature.

## Resumo

**Contexto:**

Alterações na microbiota intestinal podem estar envolvidas na fisiopatologia da alergia ao leite de vaca. No entanto, atualmente não se sabe se anormalidades na microbiota intestinal podem contribuir para a confirmação diagnóstica da alergia ao leite de vaca por meio de assinaturas específicas do microbioma.

**Objetivo:**

realizar uma revisão sistemática da literatura sobre a microbiota intestinal de lactentes com alergia ao leite de vaca.

**Métodos:**

Esta revisão sistemática incluiu estudos sobre a microbiota intestinal de lactentes menores de 2 anos com alergia ao leite de vaca no momento do diagnóstico e no acompanhamento após diferentes intervenções no controle das manifestações clínicas, em comparação com controles saudáveis. A base de dados PubMed foi utilizada para a busca bibliográfica. Foi aplicado o protocolo Preferred Reporting Items for Systematic Reviews and Meta-Analyses (PRISMA). Esta revisão foi registrada na plataforma PROSPERO (CRD42024574354).

**Resultados:**

Foram identificados 1.096 artigos. Dezoito estudos foram selecionados para revisão sistemática após a aplicação dos critérios de inclusão e exclusão. Quanto ao tipo de manifestação clínica, foram incluídos lactentes com alergia ao leite de vaca mediada por IgE (n=7), lactentes com alergia ao leite de vaca não mediada por IgE (n=10) ou ambas (n=1). O teste de provocação oral para diagnóstico de alergia ao leite de vaca foi mencionado em 11 estudos, sendo que em 7 deles foi utilizado o teste de provocação duplo-cego controlado por placebo. A maioria dos estudos (n=13) utilizou o sequenciamento do gene 16S rRNA para investigar a microbiota intestinal e apenas 3 estudos utilizaram a análise metagenômica *shotgun*. Observou-se grande heterogeneidade na expressão dos resultados quanto às características da microbiota. A alfa diversidade foi semelhante à do grupo controle na maioria dos estudos. Menor abundância de *Bifidobacteria* foi observada em alguns estudos (n=5).

**Conclusão:**

os resultados desta revisão sistemática não permitiram a identificação de um padrão típico de microbiota em lactentes com alergia ao leite de vaca. Foi também demonstrado que são necessários estudos que incluam lactentes antes da dieta de eliminação e com diagnóstico confirmado por teste de provocação oral, além de estudos que incluam um grupo de lactentes da mesma idade em aleitamento materno exclusivo e outro grupo, também da mesma idade, em aleitamento artificial como grupo controle. Portanto, os dados atualmente disponíveis não permitem estabelecer o diagnóstico de alergia ao leite de vaca por meio de uma assinatura da microbiota.

## INTRODUCTION

Cow milk allergy (CMA) is the most common food allergy in infants^1^ ^-^ ^3^. The prevalence of food allergies has increased in recent decades^2^. There are three types of CMA: immunoglobulin E (IgE)-mediated, non-IgE-mediated, and mixed. CMA is characterized by various clinical phenotypes, with common and non-specific symptoms that can occur as part of the normal development of the digestive system in infants or as other diseases of the gastrointestinal tract^1^ ^,^ ^2^. There are also concerns about a significant number of CMA overdiagnoses^1^ ^,^ ^3^, which leads to unnecessary consumption of hypoallergenic infant formulas, with a high impact on public health service expenditures or on family budget^3^. However, CMA underdiagnosis can cause physical suffering and growth deficits in infants who are not identified and diagnosed early. This complex clinical scenario is explained by the non-specific nature of the clinical manifestations and lack of accurate tests for CMA diagnostic confirmation^1^ ^-^ ^3^. Therefore, in practice, a diagnostic elimination diet followed by an oral challenge test is the most reliable method of managing infants with suspected CMA^1^ ^,^ ^2^. Despite being considered the gold standard, the oral challenge test can yield false-positive or false-negative results if performed after the development of oral tolerance.

Alterations in the gut microbiota may be involved in the pathophysiology of CMA^4^. Abnormalities in the gut microbiota can cause immune system dysregulation, resulting in inflammatory processes in the gastrointestinal tract and a predisposition to food allergies^4^. However, to date, the pathophysiological mechanism by which the gut microbiota influences the development of CMA and food tolerance remains unclear^5^. Different gut microbiota profiles have been observed in patients with IgE and non-IgE CMA compared with those in healthy children^6^. However, whether gut microbiota abnormalities contribute to the diagnostic confirmation of CMA through specific microbiome signatures is still unknown. Therefore, this study aimed to perform a systematic review of the literature on the gut microbiota of infants with CMA.

## METHODS

This systematic review was performed according to the Preferred Reporting Items for Systematic Reviews and Meta-Analyses protocol^7^, and registered on the PROSPERO platform (CRD42024574354) available at https://www.crd.york.ac.uk/prospero/display_record.php?ID=CRD42024574354.

The PICO (Patient/Population, Intervention, Comparison, and Outcome) tool was used to structure the study question: Do infants with (P) and without (C) CMA/food allergy (C) present with differences (O) in their intestinal microbiota (I)?

The PubMed (National Library of Medicine) database was used for literature search, with no language restrictions. Articles available until September 21, 2023 were included. The following search strategy was used in this systematic review: (“intestinal flora”[Title/Abstract] OR “intestinal microorganism”[Title/Abstract] OR “intestinal bacterium”[Title/Abstract] OR “intestinal flora”[Title/Abstract] OR “enteric flora”[Title/Abstract] OR “microflora”[Title/Abstract] OR “gastrointestinal microbiome”[Title/Abstract] OR “microbiome”[Title/Abstract] OR “dysbiosis”[MeSH Terms]) AND (“OPR”[All Fields] AND “dysbacteriosis”[Title/Abstract]) OR “microbiota”[Title/Abstract] OR “microbiome”[Title/Abstract]) AND (“food hypersensitivity”[MeSH Terms] OR “cow s milk allergy”[Title/Abstract] OR “food allergy”[Title/Abstract] OR “hypersensitivity”[Title/Abstract] OR “milk allergy”[Title/Abstract] OR “cow milk hypersensitivity”[Title/Abstract] OR “food protein”[Title/Abstract] OR “food protein induced”[Title/Abstract] OR “Proctocolitis”[Title/Abstract] OR “hematochezia”[Title/Abstract]).

Inclusion criteria for article selection were as follows: comparative evaluation of the intestinal microbiota of infants aged <2 years with CMA diagnosed using clinical or laboratory criteria with that of healthy controls (infants without CMA or other allergies). Exclusion criteria were as follows: animal studies, review studies, meta-analyses, abstracts of editorial meetings, duplicate studies, studies not available in full, and studies in which isolated data from patients with CMA were unavailable.

Article selection and analysis were independently performed by two authors (JSSM and MDS). In cases of disagreement between reviewers, a third reviewer (MBM) was consulted and final decision was reached by consensus. The Rayyan web application developed by the Qatar Computing Research Institute was used for study selection process and to record decisions.

A standardized Excel spreadsheet form was used to register the following data from all included articles: author, year of publication, country, study design and methodology, sample size, sex, age, diagnostic method for CMA, non-IgE-mediated clinical presentation, microbiota analysis methodology, and time of collection of stool sample for microbiota analysis (before CMA onset, during clinical manifestations before and after the start of elimination diet, and type of hypoallergenic formula).

## RESULTS

**FIGURE 1:**
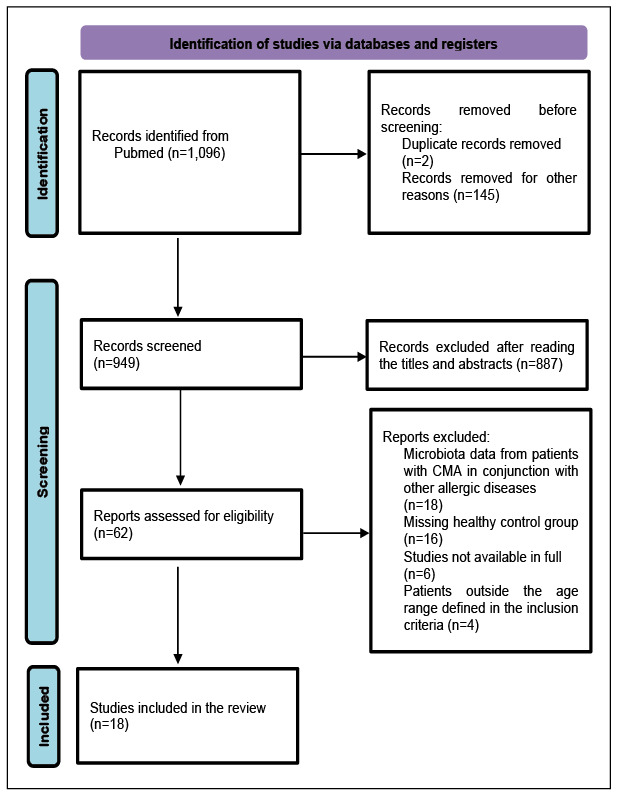
PRISMA flow diagram of selected studies for the systematic review. PRISMA: preferred reporting items for systematic reviews and meta-analyses.

A total of 1,096 articles were identified from the PubMed database of the National Library of Medicine. Two duplicate and 145 articles were removed due to various reasons (animal studies, review articles, meta-analyses, conference abstracts, editorials, duplicate studies, and studies in which data isolated from patients with CMA were unavailable). Of the remaining 947 articles, 62 were selected based on the inclusion criteria established after reading titles and abstracts. After reading the 62 selected articles in full, 44 were excluded (18 studies that presented microbiota data from patients with CMA in conjunction with other allergic diseases, 16 studies without healthy controls, 6 studies that were not available in full even after contacting the authors, and 4 studies that included patients outside the age range defined in the inclusion criteria). Finally, 18 studies were selected for the systematic review (Figure 1).

Main findings regarding the intestinal microbiota of infants with active clinical symptoms of CMA compared with those in controls are shown in Table 1, and results regarding follow-up of the intestinal microbiota with different interventions for controlling the clinical manifestations of CMA are shown in TABLE 2.

**TABLE 1:** Intestinal microbiota of infants with active clinical symptoms of CMA compared with that of controls.

Author, year, city, country	Participants and CMA diagnosis	Intestinal microbiota in infants with CMA compared with that in controls (method and results)
**Arvola (2006)** ^16^	Forty infants with rectal bleeding (mean age = 2.7 months), 68% fully breastfeed.	FISH
**Tampere, Finland**	Colonoscopy and biopsy (n=39): 84% focal erythema and/or ulceration. Inflammation in 59% and eosinophilic infiltration in 23%.	↓*Bifidobacterias;* ↓*Lactobacilos/Enterrococcus;* ≈ *Clostridium;* ≈ *Bacteroides;* ↓Total microbial count
	Cow milk elimination diet was prescribed randomly for 19 infants, while 21 remained with the same diet. Rectal bleeding resolved in approximately 5 days in all the infants, independently of diet type.	
	Open oral food challenge was positive only in 5/7 infants. According to the authors, CMA was diagnosed in only 7 (18%) infants.	
	Sixty-four healthy infants (mean age = 3.6 months) as control group.	
**Thompson-** **Chagoyan (2010 and 2011)** ^8^ ^,^ ^9^	Forty-six infants aged 2-12 months (median = 6 months) were diagnosed with IgE-mediated CMA.	FISH
**Granada, Spain**	Clear history of immediate hypersensitivity, positive skin-prick test, specific IgE to any of the cow milk protein, and positive challenge test.	Thompson-Chagoyan et al., 2010; ↑Anaerobic bactéria; ≈ Aerobic bacteria; ≈ Enterobacteria; ≈ *Lactobacilli;* ≈ *Bifidobacteria;* ≈ *Clostridia;* ↓Yeast; ↑Total microbial count; Thompson-Chagoyan et al., 2011: ≈ *Bifidobacterium genus;* ↑*Clostridium coccoides;* ↑*Atopobium cluster;* ≈ *C. leptum* group; ≈ *Bacteroides* group; ≈ *Enterobacteria* group; ≈ *Lactobacillus* group; ≈ *Streptococcus* group; ≈ *C. perfringens* + *C. difficile;* ↑Total microbial count
	Forty-six healthy (non-allergic) infants age-matched as a control group.	FISH
**Francavilla R et al., 2012** ^10^	Sixteen infants with IgE-mediated CMA (median age = 7.5 months);	↓*Bifidobacteria*s↑ ↑*Bacteroides* ↑*Clostridium*
**Bari, Italy**	Twelve healthy controls (median age = 6.9 months);	
**Kumagai H et al., 2012** ^11^	Fifteen infants with blood-streaked stools (median age = 3 months);	PCR amplification of the 16S rRNA gene
**Morioka, Japão**	Fifteen healthy controls (median age = 3 months);	↓ counts of obligate anaerobes; *↓C. leptum* *↓ Bacteroides fragilis* *↓ Prevotella* *↓ Clostridium* and *Bacteroides* *↓ E. coli* ↑*Klebsiella* *↓ C. coccoides* ≈ *Bifidobacterium* ≈ *Atopobium*
**Berni-Canani (2016)** ^12^	Nineteen infants with IgE-mediated CMA still receiving cow milk protein at age 1-12 months (mean = 4 months in an EHF group and mean = 4.3 months in an EHF + LGG group).	16S rDNA sequencing using the Illumina MiSeq platform
**Naples, Italy**	Diagnosis of IgE-mediated CMA based on clinical history, DBPCFC, and serum-specific anti-cow milk protein IgE.	↑ Shannon diversity and Pielou’s evenness ≈ abundance *↓ Bifidobacteriaceae* *↓ Streptococcaceae* *↓ Enterobacteriaceae* (↓ *Escherichia*) *↓ Enterococcaceae* ↑*Ruminococcaceae* ↑ *Lachnospiraceae* 73% *Bacteroidetes* and *Firmicutes* taxa ↑*Ruminococcus* genus ↑*Faecalibacterium* genus *↓ Bifidobacterium* *↓ Escherichia*
	Twenty healthy controls (mean age = 4.2 months)	
**Berni-Canani (2018)** ^18^	Twenty-three infants (mean age = 11.4 months) with non-IgE-mediated CMA still receiving standard formula.	V3-V4 region of the 16S rRNA sequencing using the Illumina platform
**Naples, Italy**	Diagnosis of non-IgE-mediated CMA based on clinical history, negative result of skin prick test, and/or negative level of IgE serum-specific anti-cow milk proteins, and a DBPCFC. Twenty-three infants (mean age = 12.9 months) with negative clinical history for any allergic condition (consultations for evaluation of minimal surgical procedures or vaccination programs).	≈ Shannon diversity; Phyla and genera in parentheses: ↑Bacteroidetes (↑*Bacteroides* and ↑*Alistipes*) ≈ *Firmicutes* (↑Sarcina, ≈*Lactobacillus*, and ≈*Clostridium*) ≈ *Proteobacteria* ≈ *Verrucomicrobia* ≈ *Actinobacteria* (≈*Bifidobacteria*)
**Dong (2018)** ^25^	Sixty infants ≤4 months of age (mean = 2.9 months) with challenge-proven non-IgE- or IgE-mediated CMA	16S rRNA gene sequencing using the Illumina platform
**Shanghai, China**	Clinical and laboratorial data followed by positive double-blind placebo-controlled challenge.	↓ Shannon diversity; *↓* Total microbiota ≈ *Actinobacteria* (≈ *Bifidobacteriacea*) *↓ Bacteroidetes* (↓ *Bacteroidaceae*) ↓ *Bacteroides* genus ≈ *Firmicutes* (*Lactobacillaceae; Veillonellaceae; Lachnospiraceae; Ruminococcaceae;* and ≈ class *Clostridia*) ↑*Proteobacteria* (*≈Alcaligenaceae*; ↑*Enterobacteriaceae*) ↑*Enterobacteriacea/Bacteroidaceae;*
	Sixty healthy age-matched infants exclusively breast fed until introduced to milk formulae at the same age as paired CMA infants	
**Aparicio (2020)** ^22^	Eleven infants (age ≤6 months) with non-IgE mediated CMA; 4 with regurgitation, vomiting, refusal of food, constipation, perianal erythema, and improvement with cow milk protein exclusion diet; and 3 with proctocolitis in exclusive breast feeding (no dietary intervention in the mother) and negative stool microbiological study.	16S rDNA gene using the Illumina platform
**Madrid, Spain**	No mention of oral challenge test	≈ Richness and Shannon index; Beta diversity did not reveal distinct microbial profile.
	Eight healthy controls (age ≤4 months), 7 born via vaginal delivery, and no mention of feeding type.	↓ *Bifidobacterium* ≈ *Collinsella* ≈ *Bacteroides* ≈ *Enterococcus* ≈ *Lactobacillus* ≈ *Streptococcus* *↓ Erysipelatoclostridium* ≈ *Veillonella* ≈ *Escherichia_Shigella* ≈ *Klebsiella* ≈ Unclassified_*Enterobacteriaceae* ↑*Lachnospiraceae* ↑*Eggerthellaceae* ↑*Peptostreptococcaceae*
**Mennini (2021)** ^13^	Fourteen infants aged 10-15 months (mean age = 12.9 months) with IgE-mediated CMA.	16S rRNA gene sequencing using THE Illumina platform and qRT-PCR
**Rome, Italy**	Twelve infants (mean age = 12.6 months) sensitized but tolerant to cow milk protein.	≈ Alpha-diversity (Observed, Chao1 and Shannon indexes) ≠ Beta-diversity (CMA group was clearly separated from others)
	Diagnosis of CMA: DBPCFC	↓ Phylum *Verrocomicrobia* ↑Phylum *Firmicutes (Sensibilization > healthy)* ↑Genera *Pasteurellaceae;* ↑Genera *Actinobacillus* ↑Genera *Haemophilus* ↑Genera *Erwinia* ↑Genera *Klebsiella* ↑*Prevotella* *↓ Parabacteroides* *↓* Genera *Granulicatella* ↑Genera *Streptococcus* *↓* Genera *Anaerotruncus* *↓* Genera *Ruminococcaceae_g_* ≈ *B. breve*, ≈ *B. longum subsp. Longum,* ≈ *B. longum subsp. infantis*
	Definition of sensitization: positive skin prick test and/or allergen-specific IgE.	
	Fourteen heathy controls infants (mean age = 12,6 months)	
**Martin et al. (2022)** ^23^	Eighty-one infants (mean age = 1 month) from a sample of 954 (81/954) with followed since birth; they presented with suspected FPIAP during longitudinal follow-up.	16S rRNA gene sequencing using the Illumina platform
**Boston Metropolitan area, USA**	Each physician’s clinical diagnosis of FPIAP was based on guaiac positive or grossly bloody stool not attributable to another etiology. Treatment of FPIAP was per clinician discretion. Therefore, reintroduction of the suspected offending food to confirm diagnosis was not routinely done, and the order of foods eliminated was not standardized. Information concerning symptoms resolution was collected retrospectively based on chart review.	Pre-symptomatic ≈ alpha-diversity (Chao1), except at 4 months (↑richness in symptomatic group) ≈ stability (Bray-Curtis)
	Some infants received *Lactobacillus*-containing probiotics.	0 to 2 months ↑Genera *Enterobacteriaceae* (most likely, *Escherichia*) *↓* Family *Clostridiaceae* *↓* Unknown family *Clostridiales* *↓ Lactobacillus* Last pre-symptomatic sample ↑Genera *Enterobacteriaceae*
	Seventy-nine healthy infants were paired according to type of delivery and feeding and previous use of antibiotic.	First symptomatic sample and symptomatic ↑*Enterobacteriaceae* genus *↓* family *Clostridiales;* Symptomatic ≈ alpha-diversity: Chao1 richness index *↓ Clostridiales* ↑Genera *Enterobacteriaceae (most likely, Escherichia)*
**Wurm (2023)** ^24^	Fourteen infants (mean age = 11.1 weeks) with suspected FPIAP. All recovered with an elimination diet.	16S rRNA sequencing using the Illumina platform
**Graz, Austria**	Positive oral challenge test in 2 infants.	≈ Alpha diversity index (richness, phylogeny, and Shannon uniformity) ≠ Beta-diversity: suspected FPIAP group was clearly separated from healthy controls (Bray-Curtis index and weighted UniFrac)
	Fifty-five healthy control infants (mean age = 12.7 weeks)	↓ *Genus Bifidobacterium* *↓ Bifidobacterium spp* *↓ Bifidobacterium longum* ↑*Clostridium sensu stricto* 1 ↑*Clostridium butyricum*

↑ Increase compared with the control (*P*<0.05). ↓ Reduction in relation to control (*P*<0.05). ≈ No statistically significant difference (P>0,05). CMA: cow milk allergy. DBPCFC: double-blind placebo-controlled food challenge test. EHF: extensively hydrolyzed formula. LGG: *L. rhamnosus* GG. FISH: fluorescence *in situ* hybridization. IgE: immunoglobulin E. qRT-PCR: quantitative real-time reverse transcription polymerase chain reaction. PCR: polymerase chain reaction. FPIAP: Food Protein-Induced Allergic Proctocolitis.

Regarding the type of clinical manifestation of CMA, seven studies included infants with IgE-mediated CMA^8^ ^-^ ^14^, 10 included infants with non-IgE-mediated CMA^11^ ^,^ ^16^ ^-^ ^24^, and one included both^25^.

The oral challenge test for CMA diagnosis was mentioned in 11 studies^8^ ^-^ ^10^ ^,^ ^13^ ^,^ ^16^ ^,^ ^18^ ^,^ ^19^ ^,^ ^23^ ^-^ ^25^, and in seven of them, a double-blind placebo-controlled challenge test was used^8-10,12-13,18,25^ (Tables 1 and 2).

**TABLE 2:** Follow-up of intestinal microbiota with different dietary interventions for controlling CMA.

Author, year, city, country, and participants	Intestinal Microbiota (method, intervention, and follow-up)
**Arvola (2006)** ^16^ **Tampere, Finland** Forty infants with rectal bleeding (mean age = 2.7 months), 68% fully breastfeed; Sixty-four healthy infants (mean age = 3.6 months) as control group. Rectal bleeding resolved in approximately 5 days in all the infants, independently of the diet type. The open challenge test was positive only in 5/7 infants. According to the authors, CMA was diagnosed in only seven (18%) infants.	FISH Intervention: Cow milk elimination diet (amino acid-derived formula) was prescribed randomly for 19 infants in a rectal bleeding group while 21 remained with the same diet. Assessment of the intestinal microbiota before and 30 days after starting elimination diet of cow milk protein in patients or controls: ≈*Bacteroides* ≈*Bifidobacteria* ≈*Clostridium* ≈*Lactobacilli* ≈*Enterococc*i
**Thompson-Chagoyan (2010)** ^9^ **Granada, Spain** Forty-six infants with IgE-mediated CMA; Forty-six healthy (non-allergic) infants age-matched as control group.	FISH Intervention: Infants with CMA: EHF without pre- or pro-biotics for 6 months. Healthy controls: continued to receive a cow milk formula. Assessment of the intestinal microbiota at 6 months of follow-up in infants with CMA compared with that in controls: ↑counts of anaerobic bacteria ≈ counts of aerobic bacteria ≈ counts and ↓ proportion of *Enterobacteria* ↑counts and ↑ proportion of *Lactobacilli* ↓ counts and ↓ proportion of *Bifidobacteria* ≈ counts of *Clostridia* ≈ counts and ↓ proportion of yeast ≈ total count Assessment of the intestinal microbiota at baseline and 6 months of follow-up among infants with CMA: ≈ counts of anaerobic bacteria ≈ counts of aerobic bacteria ↑counts and proportion of *Lactobacilli* ↓ counts and proportion of *Enterobacteria* ↓ counts and proportion of *Bifidobacteria* ≈ counts of *Clostridia* ≈ counts and proportion of yeast ≈ total count
**Francavilla (2012)** ^10^ **Bari, Italy** Sixteen infants with IgE-mediated CMA (median age = 7.5 months); Twelve healthy controls (median age = 6.9 months);	FISH Intervention: Infants with CMA: hydrolyzed and ultra-filtered whey protein formula (EHF) without lactose for 2 months in phase 1, followed by administration of EHF containing 3.8% lactose for an additional 2 months in phase 2. Healthy controls: none Assessment of the intestinal microbiota at the end of lactose-restricted diet (phase 1) in infants with CMA compared with that in controls: ↓ *Bifidobacteria* ↑*Bacteroides* ↑*Clostridium* ↑*Bacteroides/Prevotella* ↑*Eubacterium rectale/Clostridium coccoides* ↓ *Lactobacillus/Enterococcus* ↓ *Streptococcus/Lactococcus group* ↑*Escherichia coli* ↑SRB ↑*Atopobium group* *≈ Coriobacterium group* *≈ Clostridium histolyticum* *≈ Clostridium lituseburense* ↓ *Staphylococci* ↓ *Micrococci* Assessment of the intestinal microbiota at the end of lactose-containing diet (phase 2) in infants with CMA compared with that in controls: ↓ *Bifidobacteria* ↑*Bacteroides* ↑*Clostridium* ↑*Bacteroides*/*Prevotella* ≈ *Eubacterium rectale*/*Clostridium* coccoides ≈ *Lactobacillus*/*Enterococcus* ≈ *Streptococcus*/*Lactococcus group* ↑*Escherichia coli* ≈ SRB ↑*Atopobium group* ≈ *Coriobacterium group* ≈ *Clostridium* histolyticum ≈ *Clostridium* lituseburense ≈ *Staphylococci* ≈ *Micrococci* Assessment of the intestinal microbiota at the end of lactose-restricted diet (phase 1) in infants with CMA compared with that at the end of the lactose-containing diet (phase 2) in infants with CMA: Infants with CMA having lactose-restricted diet ↓ *Bifidobacteria* ↑*Bacteroides* ↑*Clostridium* ↑*Bacteroides*/*Prevotella* ↑*Eubacterium rectale*/*Clostridium* coccoides ↓ *Lactobacillus*/*Enterococcus* ↓ *Streptococcus*/*Lactococcus* ↑*Escherichia coli* ↑SRB ↑*Atopobium* ≈ *Coriobacterium group* ≈ *Clostridium* histolyticum ≈ *Clostridium* lituseburense ↓ *Staphylococci* ↓ *Micrococci*
**Berni-Canani (2016)** ^12^ **Naples, Italy** Nineteen infants with IgE-mediated CMA Twenty healthy controls Acquisition of tolerance (evaluated by double-blind placebo-controlled oral food challenge) following 12 months of treatment: Five of 12 (42%) in the EHF + LGG group Zero of 7 (0%) in the EHF group	16S rDNA sequencing using the Illumina MiSeq platform Intervention: Infants with CMA: EHF for 6 months (n=7) or EHF added with the probiotic LGG (EHF + LGG) for 6 months (n=12); Healthy controls: no intervention. Assessment of the intestinal microbiota in the EHF group at 6 months of follow-up compared with that at baseline: ↑ *Roseburia* Assessment of the intestinal microbiota in the EHF + LGG group at 6 months of follow-up compared with that at baseline: ↑*Blautia* ↑*Roseburia* ↑*Coprococcus* ↑Butyrate concentration in tolerant infants In samples with high levels of fecal butyrate levels subgroup: ↓ *Bacteroides* ↑*Faecalibacterium* ↑*Blautia* ↑*Ruminococcus* ↑*Roseburia* Assessment of the intestinal microbiota of an EHF+LGG group compared with that of an EHF group at 6 months of follow-up: ↑*Roseburia* ↑*Anaerofustis* ↑Fecal concentration of butyrate Assessment of the intestinal microbiota for tolerance (n = 5) and allergic reaction (n = 7) to cow milk proteins in the EHF + LGG group, before and after 6 months of treatment: Before treatment: There was no significantly different genera that identified infants who eventually became tolerant to cow milk. After treatment: ↑*Oscillospira* in allergic infants. Assessment of the intestinal microbiota for tolerance (n = 5) and allergic reaction (n = 7) to cow milk proteins in the EHF + LGG group at 6 months of follow-up: Beta diversity analysis (OTUs) clearly showed inter- and intra-group divergence between tolerant and allergic infants; ↑Fecal butyrate in the tolerant subgroup; ↑*Roseburia* and ↑ *Blautia* in tolerant samples with high levels of fecal butyrate;
**Berni-Canani (2018)** ^18^ **Naples, Italy** Twenty-three infants with non-IgE-mediated CMA divided into three groups: 1) Group 1- EHF at diagnosis before any dietary intervention (n=23); 2) Group 2 - patients with CMA treated with EHF for 6 months (n=9); 3) Group 3 - patients with CMA treated with EHF containing the probiotic *L. rhammosus* GG (LGG) for 6 months (n=14); 4. Control group - 23 healthy infants as control.	V3-V4 region of the 16S rRNA sequencing using the Illumina platform; Intervention CMA infants group 2: EHF for 6 months; CMA infants group 3: EHF added with the probiotic LGG (EHF+LGG) for 6 months; Healthy controls: none; Assessment of the intestinal microbiota in EHF+LGG group at 6 months of follow-up compared to controls: ≈ Bacteroidetes diversity pattern ≈ Bacteroidetes oligotype Bac8 ↓ Bacteroidetes oligotype Bac9 Assessment of the intestinal microbiota in EHF group at 6 months of follow-up compared to baseline: ↓ *Bacteroides* ↓ *Alistipes* ↓ Bacteroidetes oligotypes Bac10 and Bac12 Assessment of the intestinal microbiota in EHF+LGG group at 6 months of follow-up compared to baseline: ↓ *Bacteroides* ↓ *Alistipes* ↑Streptomyces ↓ Bacteroidetes oligotypes Bac10 and Bac12 ↑*Lactobacillus* Assessment of the intestinal microbiota in the EHF+ LGG group compared to EHF group at 6mo of follow-up: ↓ *Bacteroides* ↓ *Alistipes* ↑Lachnospira ↑*Ruminococcus* ((*Ruminococcaceae*) ↑*Oscillospira* ↓ *Dialister* ↑*Lactobacillus*
**Díaz M et al., 2018** ^19^ **Asturias, Espanha** Seventeen infants with non-IgE-mediated CMA Ten healthy controls	V3-V4 regions of the 16S rRNA gene sequencing using Illumina platform Intervention CMA group: elimination diet for all least 6 months (12 infants with EHF, 2 with soy formula and 3 with rice EHF) Controls: none Assessment of the intestinal microbiota in infants with non-tolerant CMA infants compared with that infants with tolerant CMA ↓ *Actinobacteria* (↓ *Bifidobacteria*) ↓ *Coriobacteriaceae* ↓ *Bifidobacteriaceae* ↑*Verrucomicrobia* ↑*Proteobacteria* Beta diversity analysis: clearly divergence between groups; Assessment of the intestinal microbiota in infants with non-tolerant CMA compared with that in healthy control infants: ↓ *Bacteroidaceae* ↑*Coriobacteriaceae* ↓ *Bacteroides* ↑*Clostridiales* ↑*Eubacterium fissicatena*
**Dong (2018)** ^25^ **Shanghai, China** Sixty infants with non-IgE-mediated or IgE-mediated CMA. Sixty healthy age-matched infants.	V3-V4 regions of the 16S rRNA gene sequencing using Illumina platform Intervention: CMA group: EHF or AAF according to the severity of the infant’s symptoms for 6 months; Controls: none. Assessment of the intestinal microbiota in infants with CMA compared that of healthy controls after 6 months of elimination diet/treatment: ≈ Total microbiota (Shannon diversity) ↓ *Actinobacteria* (↓ *Bifidobacteriacea*) ↓ Bacteroidetes, ≈ *Bacteroidaceae*, ↓ *Bacteroides* genus, ↓ *Bacteroides* species ≈ *Firmicutes* (↑ *Lactobacillaceae*; ≈ *Veillonellaceae*; ≈ *Lachnospiraceae*; ↓ *Ruminococcaceae*;) and ≈class *Clostridia* ≈ *Proteobacteria* ≈ *Alcaligenaceae*; ≈ *Enterobacteriaceae*) ≈ *Enterobacteriacea*/*Bacteroidaceae*
**Candy et al. (2018)** ^17^ **Wopereis et al. (2019)** ^23^ **Fox et al. (2019)** ^21^ **Reino Unido, Itália, Bélgica e Suécia** Seventy-one infants with suspected non-IgE mediated CMA randomly allocated to receive test (n=35) or control formula (n=36) Fifty-one breastfed infants as controls.	V3-V4 region of the 16S rRNA sequencing Intervention: Infants with suspected CMA: test formula (AAF with synbiotics: prebiotic fructo-oligosaccharides and probiotic *Bifidobacterium breve M-16V*) or control formula (AAF without synbiotics) for 8 weeks; Healthy controls: none; Assessment of the intestinal microbiota in infants on test formula with suspected CMA compared with that in infants on control formula at week 8: ↑alpha diversity (PD e Shannon index) ↑*Bifidobacteria* ↓ *Eubacterium rectale/Clostridium coccoides* (ER/CC) ↓ *Clostridium* histolyticum Assessment of the intestinal microbiota in infants on test formula with suspected CMA compared with that in healthy controls at week 8: ↓ *Bifidobacteria* ↑*Eubacterium rectale*/*Clostridium* coccoides (ER/CC) Assessment of the intestinal microbiota in infants on test and control formulas with suspected CMA compared with that in healthy controls at week 8: ↑alpha diversity (PD e Shannon index) Assessment of the intestinal microbiota in infants on test formula with suspected CMA compared that in infants on control formula at week 12: ↑alpha diversity (PD e Shannon index) ↑*Bifidobacteria* ↓ *Eubacterium rectale/Clostridium coccoides* (ER/CC) Assessment of the intestinal microbiota in infants on test formula with suspected CMA compared with that in infants on control formula at week 26: ↑alpha diversity (PD e Shannon index) ↑*Bifidobacteria* ↓ *Eubacterium rectale*/*Clostridium* coccoides (ER/CC) ↑*Veillonella sp* Assessment of the intestinal microbiota in infants on test and control formulas with suspected CMA compared with that in healthy controls at week 26: ↓ *Lachnospiraceae* ↓ *Ruminococcus* ↓ *Alistipes* Assessment of the intestinal microbiota in infants on test formula with suspected CMA compared with that in healthy controls at week 26: ≈ *Bifdobacterium spp* ≈ *Lachnospiraceae* spp
**Mennini (2021)** ^13^ **Rome, Italy** Fourteen infants with IgE mediated CMA Twelve infants sensitized but tolerant to CMA Fourteen heathy control infants	Region V3-V4 of the 16S rRNA gene sequencing using Illumina platform and qRT-PCR Intervention: CMA group: CM free diet, EHF and probiotic mixture (*Bifidobacterium breve M-16V, Bifidobacterium longum subsp. longum BB536, and Bifidobacterium longum subsp. infantis M-63*); T1 (7 days of probiotic intake); T2 (15 days of probiotic intake); T3 (30 days of probiotic intake); T4 (after 60 days from probiotic discontinuation); and T5 (after 90 days after probiotic discontinuation); Assessment of the intestinal microbiota in the CMA group compared with that at baseline after 6 months of treatment: Alpha-diversity (Observed, Chao1 and Shannon indexes): ≈ T1, T2, T3 and ↓T5 B. *breve* ≈ T1, T2, and T3 *B. longum subsp logum* ≈ T1, T2, and T3 *B. longum subsp infantis* ↑ T1, T2, T3, and ↓T4 Phylum Verrocomicrobia ↑ T1, T2, T3, and ↓T5 Genera *Akkermansia* ↑ T1, T2, T3, and T5 Phylum *Proteobacteria* ↑ T1, T2, T3, and T5 Genera *Sutterella* ↓ T1, T3, and T5 Phylum *Actinobacteria* ↑T1, T2, T3, and ↓ T5 Genera *Actinomyces* ↓ T1, T3, and T5 Genera *Prevotella* ↑ T1, T2, T3, T5 Genera *Streptococcus* ↓ T1, T2, T3 Genera *Enterococcus* ↓ T1, T3, T5 Genera *Blautia* ↑ T1, T2, T3, and ↓ T5 Genera *Ruminococcus* ↑ T1, T2, T3, T5
**Martin et al. (2022)** ^23^ **Boston Metropolitan area, USA** Eighty-one infants suspected FPIAP Seventy-nine healthy infants	Regium V4 of the 16S rRNA gene sequencing using Illumina platform Interventions: Suspected FPIAP group: per clinician discretion. Some infants received *Lactobacillus*-containing probiotics. Healthy infants: none. Assessment of the intestinal microbiota across the first year in overall infants compared with that at baseline: Alpha-diversity ↑ richness index (Chao1) from birth to 12 months; *Bacteroides* ↑ infants vaginally delivered; *Bifidobacterium* ↑ infants exclusively breastfed; *Lactobacillus* ↑ infants who received any probiotics in the first year (predominantly *Lactobacillus rhamnosus* GG); Assessment of the intestinal microbiota across the first year in infants with CMA compared with that in controls: ≈ overall microbial richness or stability across the first year; ↑ Genera *Enterobacteriaceae* (most likely, represented by *Escherichia*); Assessment of the intestinal microbiota in infants with CMA at first resolved sample compared with that in controls: ↑ Alpha-diversity richness index (Chao1); ↑ *Lactobacillus* (regardless of probiotic use) ↓ *Blautia;* and ↓ unknown family of the *Clostridiales* class;
**Mera-Berriatua (2022)** ^14^ **Zubeldia-Varela (2023)** ^15^ **Madrid, Spain** Thirty-four infants with IgE mediated CMA Sixteen healthy control infants	V3-V4 regions of the 16S rRNA gene were amplified using the Illumina platform Intervention: CMA group: 7 breastfed infants, 12 infants with EHF <2 weeks, 12 infants with breast milk and EHF, 3 infants with breast milk and cow’s milk formula; Controls: two breastfed infants, seven infants with cow milk formula, and seven infants with breast and cow milk formulas; Assessment of the intestinal microbiota in infants with CMA compared with that in controls at diagnosis or on elimination diet: ↓ Shannon’s diversity index; ↓ alpha diversity; ≈ Richness; ≈ beta diversity (PCoA Bray-Curtis) ↓ *Prevotella*ceae ↓ *Acidaminococcaceae*
**Wurm (2023)** ^24^ **Graz, Austria** Fourteen infants with suspected food protein induced proctocolitis; Fifty-five healthy controls infants.	16S rRNA sequencing using the Illumina platform and shotgun sequencing Intervention: Suspected food protein induced proctocolitis group: elimination diet (EHF for infants or CM free diet for the mother) for 4 weeks and then return to normal diet for >4 weeks (week 8); Healthy infants: none. Assessment of the intestinal microbiota in a group of infants with suspected food protein-induced proctocolitis at week 4 (end of elimination diet period) and week 8 (4 weeks of normal diet follow-up) compared with that in controls: ≈ alpha diversity (species richness, Shannon entropy, phylogenetic diversity, and evenness); ≈ Bray-Curtis distance (weighted non-phylogenetic distance) and weighted UniFrac (weighted phylogenetic distance); ≈ Genus *Bifidobacterium* (↓ *Bifidobacterium longum* but compensated by other *Bifidobacterium*, such as ↑ *Bifidobacterium bifidum, Bifidobacterium breve,* and *Bifidobacterium adolescentis*); ≈ *Clostridium* sensu stricto 1 (↑*Clostridium butyricum*). Observations: After dietary intervention, *Bifidobacterium* increased and *Clostridium* sensu stricto 1 decreased, showing no significant difference compared with that in control samples; Two infants diagnosed with food protein-induced allergic proctocolitis did not show a recovery of *Bifidobacterium spp* after the elimination diet.

↑ increase compared with control (*P*<0.05). ↓ reduction in relation to control (*P*<0.05) ≈ no statistically significant difference (*P*>0,05). CMA, cow milk allergy. EHF: extensively hydrolyzed formula. AAF: amino acid formula. LGG: *L. rhamnosus* GG. IgE: immunoglobulin E. FISH: fluorescence in situ hybridization. OTUs: operational taxonomic units. SRB: sulfate-reducing bacteria.

Regarding the methods used to investigate the intestinal microbiota, 14 studies used 16S rRNA gene sequencing^11^ ^-^ ^15^ ^,^ ^17^ ^-^ ^25^, seven used fluorescence in situ hybridization^8^ ^-^ ^10^ ^,^ ^16^ ^,^ ^17^ ^,^ ^20^ ^,^ ^21^, and three used the shotgun metagenomic analysis^14^ ^,^ ^15^ ^,^ ^24^ (Tables 1 and 2).

Significant heterogeneity was observed in the expression of results on microbiota characteristics. Alpha diversity was similar in the control group of most studies^13^ ^,^ ^18^ ^,^ ^22^ ^-^ ^24^ that evaluated the gut microbiota, whereas clinical manifestations were present before any intervention (Table 1). At the end of the follow-up period of the same infant group studied after different interventions, the alpha diversity was similar to that of the control group of two studies^23^ ^,^ ^25^, less diverse in three studies^14^ ^,^ ^15^ ^,^ ^24^, and more diverse in three articles^17^ ^,^ ^20^ ^,^ ^21^ (Table 2).

Regarding microbiota composition, the genus *Bifidobacteria* was the most frequently reported. A low abundance of *Bifidobacteria* was observed in five studies^10^ ^,^ ^12^ ^,^ ^16^ ^,^ ^22^ ^,^ ^24^, while in the other five studies, it was similar when compared with that in control groups^8^ ^,^ ^9^ ^,^ ^11^ ^,^ ^13^ ^,^ ^18^. A study on infants with allergic proctocolitis showed a decrease in *Bifidobacterium longum* and an increase in *Clostridium butyricum* ^24^.

When evaluating the intestinal microbiota after different interventions, an increase in *Bifidobacteria* was found in a study that used an extensively hydrolyzed formula with lactose^10^. Other three studies evaluated the same group of infants who were included in the same clinical trial to evaluate the efficacy of an amino acid formula with synbiotics (fructo-oligosaccharides plus *Bifidobacterium breve* M-16V)^17^ ^,^ ^20^ ^,^ ^25^. The main results after 8, 12, and 26 weeks are shown in Table 2, which illustrates the differences between amino acid formula interventions with and without symbiotic mixture addition. Other clinical trials evaluated the role of extensively hydrolyzed formulas containing *Bifidobacterium longum* subsp. *longum* BB536, *Bifidobacterium breve* M-16V, and *Bifidobacterium longum* subsp. *infantis* M-63^13^ (Table 2).

## DISCUSSION

This systematic review included studies on the gut microbiota of infants aged <2 years with CMA at diagnosis and at follow-up after different interventions to control clinical manifestations compared with that in healthy controls. Significant heterogeneity in the age of inclusion in the studies, diet consumed, diagnostic criteria for CMA, methods for analyzing gut microbiota, timing of stool analysis, different hypoallergenic formulas during the elimination diet, and heterogeneity in the expression of the characteristics of the microbiota were observed, thereby hindering interpretation of the results. Despite these limitations, it is possible to speculate that in some studies, CMA was associated with a lower participation of *Bifidobacteria*. However, these abnormalities may be related to artificial breastfeeding and not necessarily to CMA. Therefore, currently available data do not allow CMA diagnosis to be established through a microbiota signature.

Some studies included in this systematic review showed that infants with CMA had similar alpha diversity^13^ ^,^ ^18^ ^,^ ^22^ ^-^ ^24^ and lower abundance of *Bifidobacterium* ^10^ ^,^ ^12^ ^,^ ^16^ ^,^ ^22^ ^,^ ^24^ than those in control groups. Although some findings are concordant, most studies have not confirmed the presence of the same intestinal microbiota signature in infants with CMA. Furthermore, it was not possible to determine whether these abnormalities were related to artificial breastfeeding or CMA.

The methodologies used to analyze the gut microbiota also differed, and different taxonomic levels were evaluated across studies. Only the shotgun metagenomic database provides information on strains or species. Only three studies used this technique^14^ ^,^ ^15^ ^,^ ^24^. Consequently, information at this taxonomic level is limited.

Recent studies have suggested that an imbalance in the gut microbiota can lead to dysregulation of the immune and inflammatory systems in the gastrointestinal tract and a predisposition to food allergies^4^. Several environmental factors can modulate the gut microbiota, leading to an increased risk CMA. When analyzing the studies included in this systematic review, it was noted that there was great diversity and heterogeneity in the evaluated parameters that may influence the gut microbiota, limiting the analysis of the causal relationship between the microbiota and development of CMA.

When analyzing the age range of the studies analyzed, a wide range was observed among the groups studied, ranging from birth to a median age of 17 months. As the pattern of the intestinal microbiota changes with age, this wide age range could be a confounding factor in the interpretation of the results. From birth to approximately three years of age, the intestinal microbiota undergoes a maturation process and is being colonized by different bacteria, thereby increasing its diversity until stability is reached after three years of age^6^. The intestinal microbiota undergoes two extremely important transition phases. Soon after birth, the microbiota of a newborn is primarily composed of *Enterobacteriaceae* and *Staphylococcus*. With the introduction of breastfeeding, changes occur in the intestinal microbiota, with the establishment of bacteria of the *Bifidobacterium* genus. Subsequently, with the introduction of food, there is a replacement of the flora with dominance from bacteria belonging to the *Bacteroidetes* and *Firmicutes* phyla, such as the flora in the adult population^26^ ^,^ ^27^.

The criteria used for the CMA diagnosis were heterogeneous, and it was difficult to interpret the microbiota data. The oral challenge test was mentioned in 11 studies, and only 7 performed a double-blind, placebo-controlled oral challenge test, which is the main diagnostic method for CMA^1^. Kumagai et al.^11^ used hematochezia as a presumptive diagnosis of food protein-induced allergic proctocolitis. Arvola et al.^16^ did not analyze a subgroup diagnosed with CMA separately from other infants with hematochezia, while Wurm et al.^25^ included infants with suspected food protein-induced allergic proctocolitis (CMA was confirmed in only two infants). In contrast, in a large prospective study, CMA diagnosis was based on the criteria of each physician responsible for infant care, who was also responsible for defining management, including the type of elimination diet^23^. In this population-based cohort of 954 newborns from a Boston neighborhood, 153 (17%) were suspected to have food protein-induced allergic proctocolitis. Of the 153 infants, 81 from whom the minimum defined number of stool samples were collected were included in comparative study of the microbiota. The prevalence of food protein-induced allergic proctocolitis is very high compared with that expected for all forms of CMA, ranging from £1% to 3-5% of the population^1^ ^-^ ^4^. Therefore, it is likely that several patients suspected of having food protein-induced allergic proctocolitis did not actually have CMA because the diagnosis was not confirmed by challenge testing^23^.

Therefore, the lack of a precise diagnosis in several studies may have led to incorrect diagnoses, allowing patients with other food allergies or other conditions to be included, leading to an overdiagnosis of the disease and affecting the analysis of the clinical results.

The diet at admission was heterogeneous among infants with CMA, ranging from exclusive breastfeeding, bottle feeding, mixed feeding with cow milk, extensively hydrolyzed formula or amino acid formula, or complementary feeding. Given that each type of diet exerts a different effect on the gut microbiota, it is possible that infants respond differently to the elimination diet because the mechanism for developing tolerance is likely not the same. In the healthy control group, the infants’ diets at admission varied between exclusive breastfeeding, bottle feeding, or mixed feeding. Diet is crucial for the formation of gut microbiota, and the ideal diet for the development of a healthy microbiota is exclusive breastfeeding; a control group should comprise only exclusively breastfed infants^28^ ^,^ ^29^. Some studies have shown that the use of probiotics or synbiotics, when added to hypoallergenic formulas, helps in the development of tolerance to cow milk protein due to their immune-modulating effects; however, there is insufficient evidence in favor of the use of these formulas when compared with standard hypoallergenic formulas^5^ ^,^ ^30^ ^-^ ^32^.

The gut microbiota of infants with CMA was associated with a high abundance of the phyla *Firmicutes* (class *Clostridia*; the families *Lachnospiraceae* and *Ruminococcaceae*; the genera *Clostridium*, *Faecalibacterium*, *Lactobacillus*, *Ruminococcus*, and *Subdoligranulum*), *Bacteroidetes* (genera *Bacteroides* and *Prevotella*), and *Proteobacteria* (genera *Haemophilus*, *Actinobacillus*, and *Klebsiella*; and the species *Escherichia coli*), and regarding the phyla *Actinobacteria*, there was reduced levels in the order *Lactobacillales* and genus *Bifidobacterium* ^33^.

The genus *Bifidobacteria* was the most frequently reported in the selected studies, but a lower abundance of *Bifidobacteria* was observed in only five studies^10^ ^,^ ^11^ ^,^ ^15^ ^,^ ^21^ ^,^ ^23^, which differs from the previously described findings^33^.

A major limitation of this systematic review was the heterogeneity of the compiled articles regarding the inclusion criteria, type of diet used at admission, diagnostic criteria for CMA, and methods for assessing the intestinal microbiota.

A strength of this review was the inclusion of studies that used a control group of infants, as the intestinal microbiota changes throughout the first year of life, regardless of the diagnosis of CMA.

In conclusion, the results of this systematic review did not identify typical microbiota patterns in infants with CMA. Studies that include infants before the elimination diet and those with a diagnosis confirmed using the challenge test are needed. For a control group, the ideal would be to have one group of infants of the same age exclusively breastfed and then another of the same age fed with formula. Therefore, currently available data do not allow the diagnosis of CMA to be established through a microbiota signature.

## Data Availability

Data in article: the research data are presented within the article itself (available in Tables 1, and 2).
